# Supplementary material for: Cooperation in a fluid swarm of fuel-free micro-swimmers
Source: Nat Commun. 2022 Jan 10;13:184. doi: 10.1038/s41467-021-27870-9 (PMC8748659; doi:10.1038/s41467-021-27870-9)
Supplement: Supplementary file 1 — Supplementary Information [file 41467_2021_27870_MOESM1_ESM.pdf]

Cooperation in a fluid swarm of fuel-free micro-swimmers

Supplementary Information

Matan Yah Ben Zion*[1,2], Yaelin Caba[1], Alvin Modin[1], and Paul M Chaikin[1]

[1]Center for Soft Matter Research, Department of Physics, New York University, 726 Broadway Avenue, New York, NY 10003, USA

[2]UMR Gulliver 7083 CNRS, ESPCI Paris, PSL Research University, 10 rue Vauquelin, Paris 75005, France

Synthesis Overview

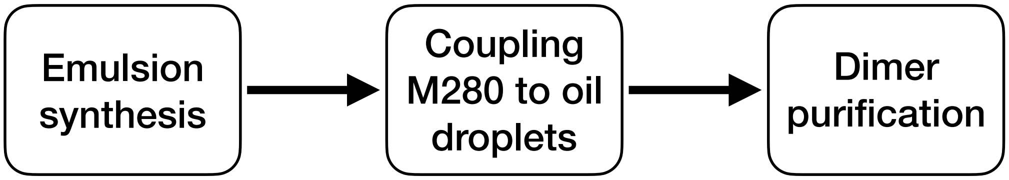

Supplementary Figure 1: Overview of synthetic steps.

Emulsion Synthesis

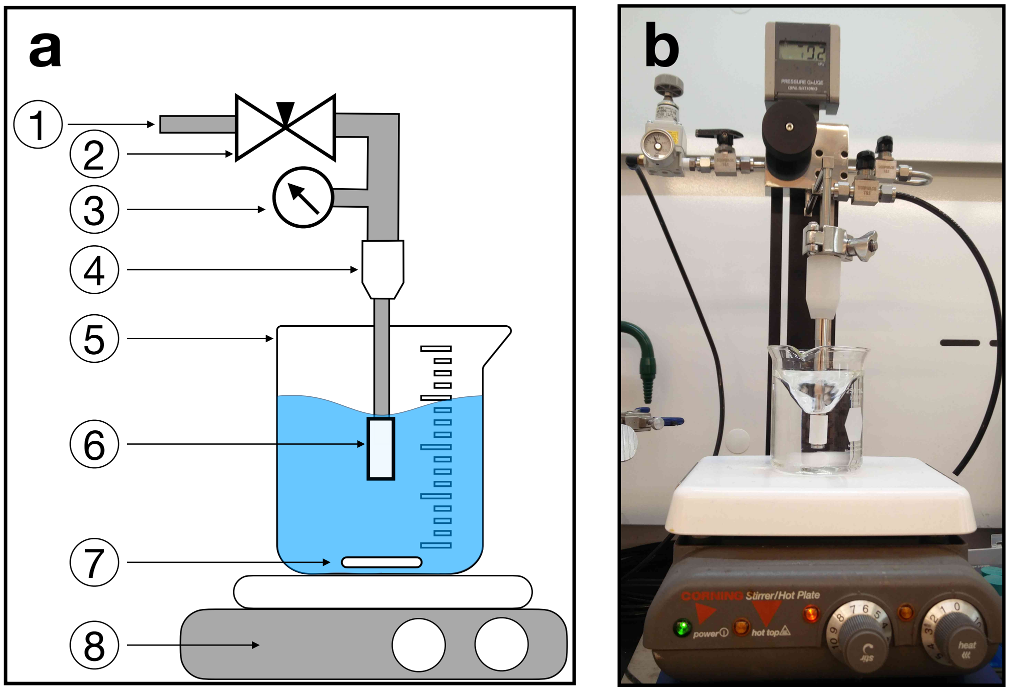

Supplementary Figure 2: Emulsion synthesis through membrane emulsification. **a** membrane emulsification diagram: 1. Air pressure point; 2. Needle valve; 3. Pressure gauge; 4. Oil container; 5. Glass beaker; 6. Porous silica membrane; 7. Magnetic stir bar; 8. Stirring plate. **b** Photo of the membrane emulsification setup.

*Correspondence and requests for materials should be addressed to M.Y.B.Z (email: matanbz@gmail.com)

Coupling Light Absorbing Particles to Liquid Droplets

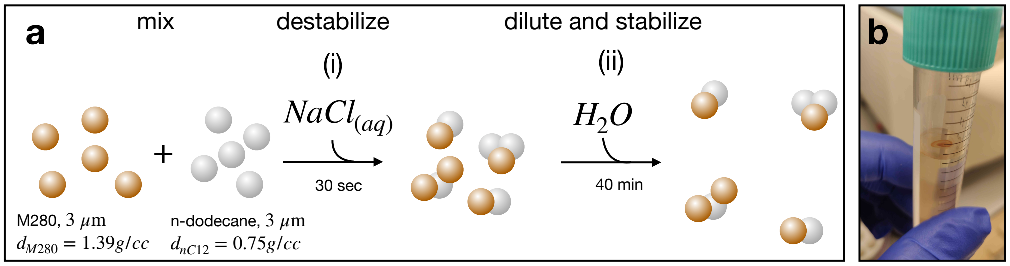

Supplementary Figure 3: Coupling light absorbing particles (M280) to fluid droplets (n-dodecane). **a** Steps in the binding process where a suspension of M280 particles and an emulsion of n-dodecane droplets are mixed, the destabalized by reducing the screening length using salt (i) then stabalized by diluting with a large volume of water (ii). **b** photo of a tube after the dilution step (the brown mist is the M280 particles).

Dimer Purification

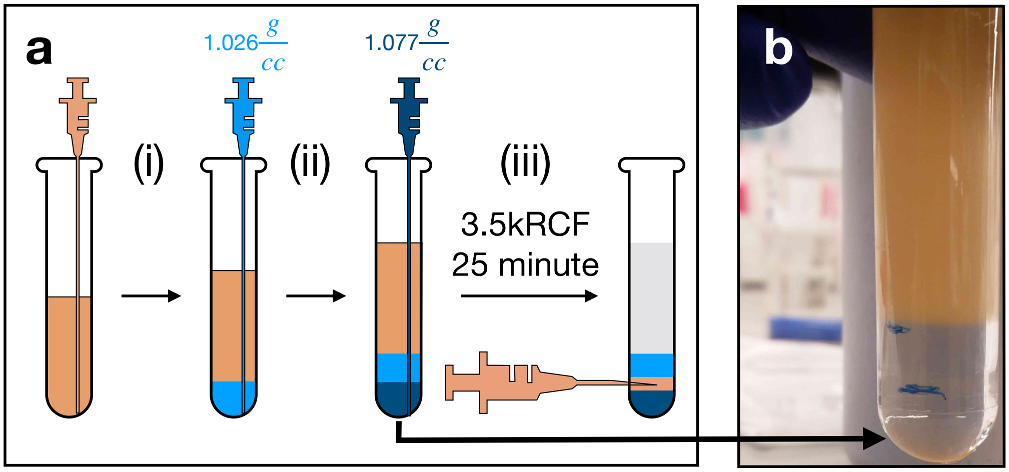

Supplementary Figure 4: Dimer purification. **a** DImers are purified by tailoring density gradient steps at (i) a slightly lower density, $1.026 g/cc$ (light blue), and (ii) a slightly higher density, $1.077 g/cc$ (dark blue) than the expected density of the dimers, $1.07 g/cc$. The centrifuge is the spun (iii) and the dimers accumlate between the two dense phases to form a visible band. **b** photo of the separation tube before centrifugation.

Experimental Setup

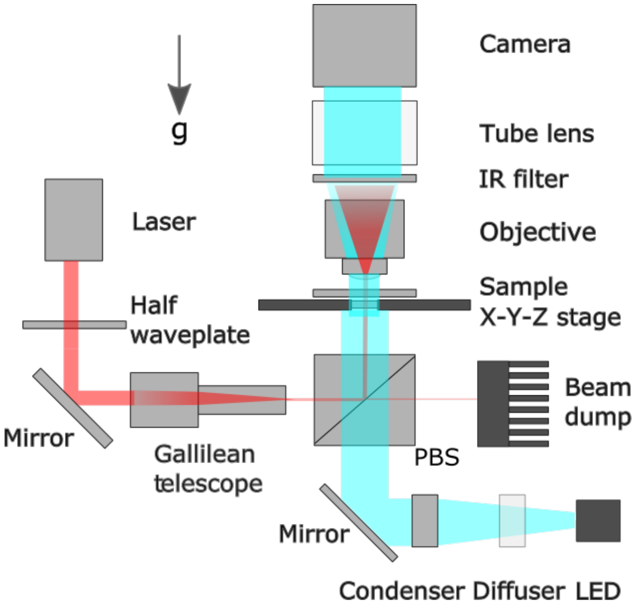

Supplementary Figure 5: Schematics of experimental setup.

1 Light Driven Thermo-Capillary Swimmer Model

1.1 Fluid Droplet in a Temperature Gradient

For the thermo-capillary swimmers presented, the temperature gradient is given by the spatial derivative of the temperature profile around the hotter light absorbing particle when it is exposed to radiation

$$\vec{\nabla} T = -\frac{P_0}{4\pi\kappa_w \mathrm{R}^2}\hat{\mathbf{e}}_{\mathbf{r}}, \tag{3}$$

where P_0 is the heating power at the particle, and R the distance from its center, along the radial direction $\hat{\mathbf{e}}_{\mathbf{r}}$. Eq. 3 shows that the temperature gradient scales as $1/R^2$. The power absorbed by the particle is given by $P_0 = \sigma J$, where J is the flux and σ the particle's absorption cross section (see section 1.3 for measurement of σ). On the length scale of the swimmers, heat transport is dominated by high thermal diffusivity, therefor the temperature profile is assumed to move with the swimmer. Swimmer-swimmer thermal interactions are kept to minimum as the thermal conductivity of the surrounding water and adjacent cover slip are 4-10 greater than that of the oil (see Table 1). This choice of materials sets the temperature gradient (Eq. 3) to be the greatest across the tightly coupled oil droplet. Since the droplet is intimately bound to the particle, we approximate the temperature gradient to be constant with its value at the center of the droplet, allowing us to use Eq. 1 in the main text. To quantitatively verify that indeed this is the correct swimming mechanism, we directly measured the thermo-capillary coefficient, β (see section 1.2), and use literature data for the thermal conductivies and viscosities of the different swimmers species (see Table 1). The swimmer's proximity to the wall, $\delta \approx 0.2$ nm (estimated from its gravitational height), leads to enhanced drag, estimated to increase by a factor of $\frac{8}{15}\log\left(\frac{\mathrm{D}}{2\delta}\right)$ for a sphere moving along a solid wall in Stokes flow[33].

Material	κ_f $\left(\frac{\mathrm{W}}{\mathrm{K\ m}}\right)$	η_f (mPas)
n-decane	0.15	0.93
n-dodecane	0.14	1.5
n-tetradecane	0.14	2.3
n-hexadecane	0.14	3.5
mineral oil	0.17 *	13 *

Table 1: Material properties of different fluid droplets were taken from the literature[57–60]. *According to manufacturer.

1.2 Thermo-Capillary Measurement

Surface tension measurements were carried out using a commercial optical tensiometer (THETA ONE ATTENSION) by hanging a deionized water drop (18 MΩcm, TOC = 8 ppb, MILLIPORE MILLI-Q) in a n-dodecane (ALPHA AESAR) bath with 0.1%v/v SPAN-80 (SIGMA) inside a glass cuvette (THERMOFISHER) and fitting the profile of the pendant drop to the Young-Laplace model[28]. The cuvette was wrapped with resistive heating wire to ensure a homogeneous temperature, monitored using a thermometer (BS4 59-7569 Monitoring Thermometer) with a thermo-couple sensor (IT-21 microprobe) placed in close proximity to the droplet. Surface tension readings were taken after pendant drop was allowed to equilibrate at each temperature. The surface tension was then measured for multiple droplets, and their average reading was taken. Temperature was varied between 21-27°C and with a surface tension increase from 5.2 $\frac{\mathrm{mN}}{\mathrm{m}}$ at room temperature, to 8 $\frac{\mathrm{mN}}{\mathrm{m}}$. The linear thermo-capillary coefficient is found from linear regression (see Fig.6) to be $\beta = 0.58 \pm 0.02\ \frac{\mathrm{mN}}{^\mathrm{o}\mathrm{Cm}}$, quantitatively consistent with known values in the literature[29–31].

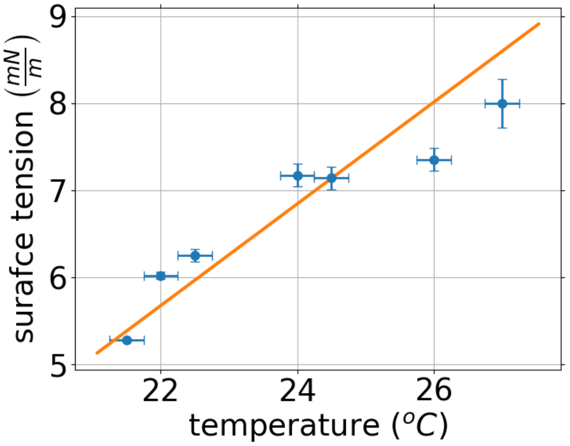

Supplementary Figure 6: Linear thermo-capillary coefficient measurement.

1.3 Laser Heating Power Balance

Heating measurements were carried out by tracking the temperature increase rate, $\frac{\partial T}{\partial t}$, (BS4 59-7569 Monitoring Thermometer) with a thermo-couple sensor (IT-21 microprobe) placed inside a standard glass cuvette (THERMOFISHER length $L = 1$ cm) filled with a suspension of $D = 3$ μm light absorbing particles (M280

Bangs Lab) in deionized water (18MΩcm, TOC=8 ppb, MILLIPORE MILLI-Q), when irradiated with a collimated 1064 nm laser beam (YLR-10-1064-LP, IPG Photonics) while stirring using a magnetic stir bar to ensure rapid thermal homogenization. The total light intensity of the beam, $P = 1.3$ W was measured using an optical power meter (PM100D power meter, with S175C sensor, THORLABS), and temperature increase rate was found from the temperature vs. time slope at early times. In order to extract the particle's absorption cross section, $\sigma = \pi\left(\frac{D}{2}\right)^2\sigma_0$ (where σ_0 is the differential cross section for absorption), the measurements were taken at increasing volume fraction, Φ ($0 - 0.4\%$v/v), eliminating the large but constant contribution to the heating, W, by the water bulk. Assuming dilute suspension ($\Phi \ll 1$), small temperature gradients from ambient ($t \ll 1$ min), and fast homogenization (stirring), the light absorbing particles contribution to the heating is given by the slope of

$$\frac{\partial T}{\partial t} = W + \frac{\frac{3}{2}\frac{L}{D}P}{C_P}\sigma_0\Phi, \tag{4}$$

where the heat capacity of the fluid, $C_P = c_p m_{\mathrm{H_2O}}$ is dominated by the water, and the differential cross-section is found to be $\sigma_0 = 5\%$, (see Fig.7). Combining our findings we expect that a swimmer moving at $5\ \mu m/s$ absorbs a net power of $0.5\ \mu W$.

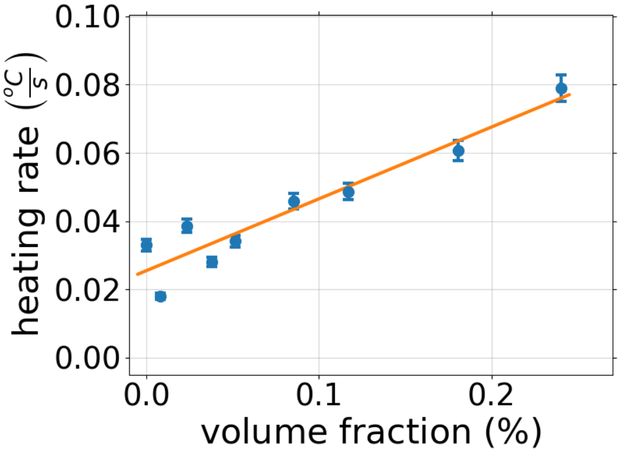

Supplementary Figure 7: Thermal heating due to light absorption

1.4 Beam Size Calibration

The beam profile was measured by exposure to infrared visualizing shavings (980nm-1064nm IR Infrared Laser Visualizer) fixed inside a UV curable resin (LOON OUTDOORS UV CLEAR FLY FINISH) between a microscope slide and a cover-slip. Laser beam intensity, I, was measured at the objective using an optical power-meter (PM100D power meter, with S175C sensor, THORLABS). The visible light emitted from the IR visualizer was imaged using a lower magnification objective (Leica HC Pl Fluotar 10X), to create the intensity profile (see Figs.5, 8). The spatial distribution of the beam $J(R)$ was then fitted to a two dimensional Gaussian $J = J_0 e^{-\mathrm{R}^2/\sigma^2}$, with R the radial distance from the Gaussian center, $J_0 = \frac{\mathrm{I}}{\pi\sigma^2}$ being the flux at the center of the beam, and $2\sigma = 310\ \mu$m is the fitted beam diameter (see Fig.8).

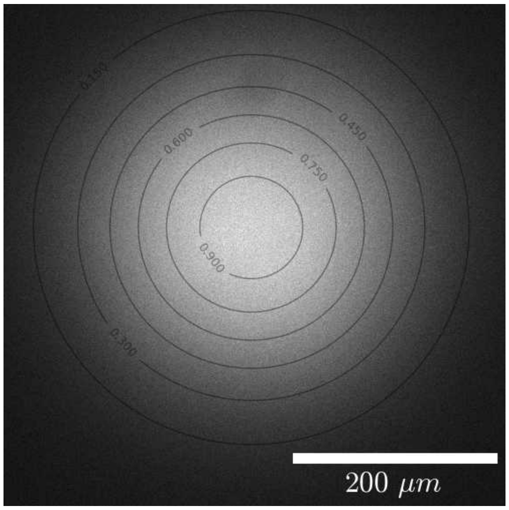

Supplementary Figure 8: Broad field image of laser beam (contours show relative power given by Gaussian fit). Experiments were performed within a field of view of $\sim$ 100 μm at the center of the beam where power spatial variation is smaller than 10%.

2 Persistent Swimmer Model

Individual swimmer's dynamics were modelled as a persistent random walk with speed v, persistent time τ, and a diffusion constant D_0. The mean square displacement, $\langle \Delta L^2 \rangle$, of such a swimmer can be found in[22]:

$$\langle \Delta L^2 \rangle = 4D_0 \Delta t + \frac{v^2 \tau^2}{2} \left[\frac{2\Delta t}{\tau} + e^{-2\Delta t/\tau} - 1 \right]. \tag{5}$$

Which for the short time limit ($\Delta t \ll \tau$) is ballistic, $\langle \Delta L^2 \rangle \approx v^2 \Delta t^2 \propto \Delta t^2$, and for longer lag times ($\Delta t \gg \tau$) behaves diffusively with an enhanced diffusion constant, $\langle \Delta L^2 \rangle \approx \left(4D_0 + v^2 \tau\right) \Delta t \propto \Delta t^1$. Swimmer videos were preprocessed using ImageJ[61], and the subsequent particle motion tracked and analyzed using Trackpy package on Python[62]. Fig.2**a** in the main text shows the Brownian ($\propto \Delta t^1$) to ballistic ($\propto \Delta t^2$) transition for increased flux. Swimming speed was independently measured by imposing their orientation using a custom built vector magnet made of 3 Helmholtz pairs, generating a constant homogenous magnetic field ($\sim$ 1mT) in the imaging plane. In the presence of a magnetic field, the swimmer maintains a fixed direction (see Fig.2**d**), allowing extraction of the swimmers speed, v, by measuring displacement over time, which is found to be linear with flux (as shown in Fig.2**b**) and is expected by the model (Eq. 1 in main text). Note that the swimming direction is not necessarily along the magnetic field as the dimmer orientation is decoupled from the magnetic dipole (for example see Fig.2**d**). We find that the reorientation time of the swimmer is then $\tau \approx 15$ s, corresponding to a persistence length of up to 70 μm with a Peclet number $Pe \approx 200$ using the definition found in the literature[8]. As our microscope imaging is done from the top, with the laser introduced from the bottom (see Supplementary Fig.5), swimmers are subjected to radiation pressure and swim on the ceiling of the capillary, explaining the small deviation from linearity at low fluxes in Fig.2**b**. We did however perform similar experiment on an inverted version of our apparatus (that is inverted imaging and laser introduced from above), finding similar swimmers dynamics.

3 Local Density Measurement

Local instantaneous density measurements were done by locating the two dimensional coordinates of all the particles in each frame (x_i, y_i, t_i)[62], around which a Voronoi tessellation was constructed using standard Python routines. The local area fraction of each particle ϕ_i is found by dividing the area of each particle, a_i by the area, A_i of its Voronoi cell $\phi_i \equiv \frac{a_i}{A_i}$. Histograming these values over 2600 frames gives the two population densities found in Fig. 3**b**. The area fraction field, $\phi(x, y, t)$ is then found piecewise from the local area fraction of each particle $\phi(x, y, t) = \sum_i \delta(x - x_i)\,\delta(y - y_i)\,\delta(t - t_i)\,\phi_i$ (where δ is the Dirac delta function). The single swimmer speed as a function of concentration $v(\phi)$ is obtained by tracking a swimmer's displacement, $|\Delta r|$, moving in the dynamic environment. Average displacement as a function of concentration $\langle|\Delta r|\rangle(\phi)$ is then found by averaging displacements within density bins of 6.7% area fraction within time intervals of 5 seconds from which points in Fig. 3**b** are measured. Error bars are the standard deviation of the displacement. A prediction for critical crowding area fraction, ϕ^*, is then found by fitting the measured displacement-concentration curve $\Delta|r|(\phi)$ to a sigmoid $f(\phi) = \frac{B}{1+e^{(\phi-\phi^*)/\alpha}} + C$ (σ, B, and C are constants) and the critical crowding concentration can be found from when satisfying the theoretically expected relation[34] of the derivative: $\frac{\partial f}{\partial \phi} = -\frac{f}{\phi}$.

4 Critical Crowding Concentrations

In order to find the critical area fraction, ϕ^*, above which swimmers form a crowded phase, a series of experiments were carried out initiating swimming in regions with a homogeneous swimmer distribution with an initial mean area fraction, ϕ_0 calculated by the total area of the observed particles, $\sum_{i=1}^{N_0} a_i$, over the area of the field of view (A_{FOV} kept constant throughout the experiment) $\phi_0 \equiv \frac{1}{A_{\mathrm{FOV}}}\sum_{i=1}^{N_0} a_i$, where N_0 the initial number of particles. As the collective dynamics evolve, with the number of swimmers inside the FOV is monitored as a function of time $N(t)$, given the evolution of the relative number $\frac{N(t)}{N_0}$ is found in Fig. 3**d**.

5 Velocity Correlation Function from Particle Image Velocimetry

The normalized velocity correlation function,

$$\left\langle \vec{V}(0) \cdot \vec{V}\left(\vec{R}\right) \right\rangle_N = \frac{1}{\left\langle \vec{V}(\vec{r})^2 \right\rangle_r} \left\langle \vec{V}(\vec{r}) \cdot \vec{V}\left(\vec{r} + \vec{R}\right) \right\rangle_r , \tag{6}$$

was found from the auto correlation of the velocity field, $\vec{V}\left(\vec{R}\right)$ ($\langle\rangle_r$ denotes spatial average). The velocity field $\vec{V}\left(\vec{R}\right)$ was found using *OpenPIV* python library[63] from pairs of frames 1 second apart taken from within the dense phase for the duration of the experiment. The VCF is computed component-wise in Fourier space $\tilde{V_i}\left(\vec{k_i}\right)$ (index i is for each coordinate $i \in [x, y]$) then using convolution theorem, converted to real space from the inverse Fourier transform of $V\tilde{C}F\left(\vec{k}\right) = \left|\tilde{V_x}\right|^2\left(\vec{k}\right) + \left|\tilde{V_y}\right|^2\left(\vec{k}\right)$, with angular averaging to extract the radial dependence.

6 Spatial Correlations of Passive Particles

Structure factor for the passive particles, $S(\vec{q})$, was defined by

$$S(\vec{q}) = \frac{1}{N} |\tilde{\rho}(\vec{q})|^2 , \tag{7}$$

where $\vec{q}$ is the wave vector, and $\tilde{\rho}(\vec{q})$ is the Fourier transform of the particle density, $\rho(\vec{r})$, which we compute by plotting $\rho(\vec{r}) = \sum_i^N F\left(\vec{R}_i\right)$, with $\{\vec{R}_i\}$ being the set of experimentally located particle coordinates, and F being a Gaussian form factor that reduces short wave length noise. See previous work for details[64,65]. The radial pair correlation function, $g(r)$ in the same figure was calculated by directly histogramming the distances, r, measured any two pairs of passive particles, with constant binning of 200 nm (0.067 particle diameter).

7 Corralling Through Excluded Area

The collective dynamics were measured by choosing a field of view with a rather homogenous particle distribution, turning the light on, and tracking the dynamics. We define the three states as follows: dispersing state where after turning on the light source, most of the particles leave the field of view (FOV); the crowding state where after turning on the light still most of the swimmers are still inside the FOV; and the corralling state where the major entity left in the FOV is the passive particles. Points on Ternary phase space are defined as described in section 4, by measuring the initial area fraction of passive ϕ_P and active ϕ_A, and plotted using *python-ternary* package[66].

To derive the criterion for corralling we note that the area available for the active particles, A, is the total area, A_T, after accounting for the effective area taken by the passive particles A_P^{eff}

$$A = A_T - A_P^{eff}. \tag{8}$$

Generally the area occupied by the passive particles depends on their spatial arrangement. Given that there are N_A active (N_P passive) particles, each of which occupies an area a_A (a_P), their area fraction is defined as $\phi_A \equiv \frac{a_A N_A}{A_T}$ ($\phi_P \equiv \frac{a_P N_P}{A_T}$). The effective area fraction of the active particles is then defined as $\phi_A^{\text{eff}} \equiv \frac{N_A}{A}$ giving

$$\phi_A^{\text{eff}} = \phi_A \Big/ \left(1 - \frac{A_P^{eff}}{A_T}\right) , \tag{9}$$

which in general also depends on the spatial arrangement of the passive particles. The question thus becomes the following: for a given spatial arrangement of the passive particles, is the effective area fraction of the active particles super critical: $\phi_A^{\text{eff}} > \phi^*$? We shall treat two cases of different spatial arrangement of the passive particles, Random, and Hexgonally Packed (HP). When the passive particles are randomly distributed, additionally to their own area, a_P, each particle occupies a corona of excluded region, inaccessible to other particles (see Figure 9**a**). For the simple case of equisized particles, this excluded area is 3 times that of the

particle itself, $a_P^e = 3a_P$, making the effective area occupied by the passive particles

$$A_P^{\mathrm{rand}} = 4a_P N_P. \tag{10}$$

If however the passive particles are arranged in a HP crystal, their effective area is considerably smaller, $A_P^{\mathrm{HP}} = \frac{\sqrt{12}}{\pi} a_P N_P + O\left(\sqrt{N_P}\right)$ (as can be seen in Fig.9**b**). Here the excluded area becomes sub-extensive as it grows like the perimeter of the crystal, and for large enough crystals, we can approximate

$$A_P^{\mathrm{HP}} \approx \frac{\sqrt{12}}{\pi} a_P N_P. \tag{11}$$

Plugging the two cases (Eqs. 10 and 11) into Eq. 9 we recover the result in the main text (Eq.1 in main text). This shows that there are combinations of concentrations of the active and passive particles, where the effective area fraction of the active particles, ϕ_A^{eff}, can either be above or below the critical crowding concentration, ϕ^*, depending on the arrangement of the passive particles. The system is expected to be in the crowding state when even if the passive particles were hexagonally packed, the effective area fraction of the active particles is still *above* critical, $\phi^{eff} = \phi_A \Big/ \left(1 - \frac{\sqrt{12}}{\pi}\phi_P\right) > \phi^*$ (red region in phase diagram in Fig. 4**a** in the main text). Correspondingly, if the passive particles were randomly distributed, yet the effective area fraction of the active particles was still *below* critical, $\phi_A^{eff} \phi_A \Big/ (1 - 4\phi_P) < \phi^*$, the system is said to be in the dispersing regime (green shade in phase diagram in Fig. 4**a**). Between these two regimes, the corralling state is defined (yellow region in Fig.4**a**).

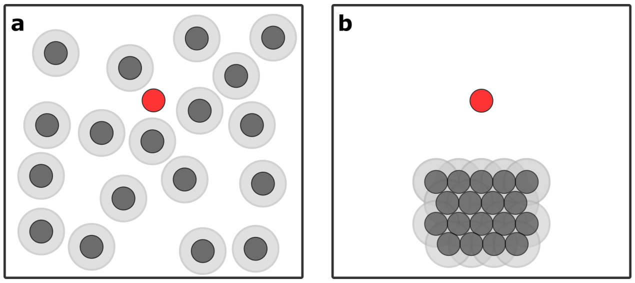

Supplementary Figure 9: Geometry of effective area occupied by 18 passive particles when **a** randomly distributed, or **b** hexagonally packed. Light shaded areas are the regions excluded for the test particle (red).

Supplementary References

[1] Hamann, H. *Swarm Robotics: A Formal Approach* (Springer International Publishing, Cham, 2018). URL `http://link.springer.com/10.1007/978-3-319-74528-2`.

[2] Procaccini, A. *et al.* Propagating waves in starling, Sturnus vulgaris, flocks under predation. *Animal Behaviour* **82**, 759–765 (2011). URL `http://dx.doi.org/10.1016/j.anbehav.2011.07.006`.

[3] BBC. Feeding Frenzy, from The Hunt: Hunger at Sea (Oceans) (2015).

[4] Harari, Y. N. *Sapiens : a brief history of humankind* (Harper, New York, 2015).

[5] Costerton, J. W., Stewart, P. S. & Greenberg, E. P. Bacterial biofilms: A common cause of persistent infections. *Science* **284**, 1318–1322 (1999).

[6] Saintillan, D. & Shelley, M. J. Instabilities, pattern formation, and mixing in active suspensions. *Physics of Fluids* **20**, 123304 (2008). URL `http://aip.scitation.org/doi/10.1063/1.3041776`.

[7] Secchi, E. *et al.* Intermittent turbulence in flowing bacterial suspensions. *Journal of The Royal Society Interface* **13**, 20160175 (2016). URL `https://royalsocietypublishing.org/doi/10.1098/rsif.2016.0175`.

[8] Marchetti, C. M. *et al.* Hydrodynamics of soft active matter. *Reviews of Modern Physics* **85**, 1143–1189 (2013). `abs/1207.2929`.

[9] Dunkel, J. *et al.* Fluid Dynamics of Bacterial Turbulence. *Physical Review Letters* **110**, 228102 (2013). URL `https://link.aps.org/doi/10.1103/PhysRevLett.110.228102`. `abs/1302.5277`.

[10] Wensink, H. H. *et al.* Meso-scale turbulence in living fluids. *Proceedings of the National Academy of Sciences* **109**, 14308–14313 (2012). `abs/1302.5277`.

[11] Zhang, H. P., Be'er, A., Florin, E. L. & Swinney, H. L. Collective motion and density fluctuations in bacterial colonies. *Proceedings of the National Academy of Sciences of the United States of America* **107**, 13626–13630 (2010).

[12] Dombrowski, C., Cisneros, L., Chatkaew, S., Goldstein, R. E. & Kessler, J. O. Self-Concentration and Large-Scale Coherence in Bacterial Dynamics. *Physical Review Letters* **93**, 098103 (2004).

[13] Xu, H., Dauparas, J., Das, D., Lauga, E. & Wu, Y. Self-organization of swimmers drives long-range fluid transport in bacterial colonies. *Nature Communications* **10**, 1–12 (2019). URL `http://dx.doi.org/10.1038/s41467-019-09818-2`.

[14] Gompper, G. *et al.* The 2020 motile active matter roadmap. *Journal of Physics: Condensed Matter* **32**, 193001 (2020). `abs/1912.06710`.

[15] Van Der Linden, M. N., Alexander, L. C., Aarts, D. G. & Dauchot, O. Interrupted Motility Induced Phase Separation in Aligning Active Colloids. *Physical Review Letters* **123**, 98001 (2019). URL `https://doi.org/10.1103/PhysRevLett.123.098001`. `1902.08094`.

[16] Geyer, D., Martin, D., Tailleur, J. & Bartolo, D. Freezing a Flock: Motility-Induced Phase Separation in Polar Active Liquids. *Physical Review X* **9**, 31043 (2019). URL `https://doi.org/10.1103/PhysRevX.9.031043`. `1903.01134`.

[17] Bäuerle, T., Fischer, A., Speck, T. & Bechinger, C. Self-organization of active particles by quorum sensing rules. *Nature Communications* **9**, 3232 (2018). URL `http://dx.doi.org/10.1038/s41467-018-05675-7http://www.nature.com/articles/s41467-018-05675-7`.

[18] Yan, J. *et al.* Reconfiguring active particles by electrostatic imbalance. *Nature Materials* **15**, 1095–1099 (2016).

[19] Lozano, C., ten Hagen, B., Löwen, H. & Bechinger, C. Phototaxis of synthetic microswimmers in optical landscapes. *Nature Communications* **7**, 12828 (2016). URL `http://www.nature.com/articles/ncomms12828`.

[20] Maass, C. C., Krüger, C., Herminghaus, S. & Bahr, C. Swimming Droplets. *Annual Review of Condensed Matter Physics* **7**, 171–193 (2016). URL `http://www.annualreviews.org/doi/10.1146/annurev-conmatphys-031115-011517`.

[21] Izri, Z., van der Linden, M. N., Michelin, S. & Dauchot, O. Self-Propulsion of Pure Water Droplets by Spontaneous Marangoni-Stress-Driven Motion. *Physical Review Letters* **113**, 248302 (2014). URL `https://link.aps.org/doi/10.1103/PhysRevLett.113.248302`.

[22] Howse, J. R. *et al.* Self-Motile Colloidal Particles: From Directed Propulsion to Random Walk. *Physical Review Letters* **99**, 048102 (2007). URL `https://link.aps.org/doi/10.1103/PhysRevLett.99.048102`. `0706.4406`.

[23] Palacci, J., Sacanna, S., Steinberg, A. P., Pine, D. J. & Chaikin, P. M. Living Crystals of Light-Activated Colloidal

Surfers. *Science* **339**, 936–940 (2013). URL http://www.sciencemag.org/cgi/doi/10.1126/science.1230020.

[24] Ben Zion, M. Y., Caba, Y., Sha, R., Seeman, N. C. & Chaikin, P. M. Mix and match - A versatile equilibrium approach for hybrid colloidal synthesis. *Soft Matter* **16**, 4358–4365 (2020).

[25] Young, B. N., Goldstein, J. S. & Blocks, M. J. The motion of bubbles in a vertical temperature gradient. *Journal of Fluid Mechanics* **6**, 350–356 (1959).

[26] Barton, K. D. & Shankar Subramanian, R. The migration of liquid drops in a vertical temperature gradient. *Journal of Colloid And Interface Science* **133**, 211–222 (1989).

[27] Doi, M. *Soft Matter Physics* (Oxford University Press, Oxford, 2013).

[28] de Gennes, P.-G., Brochard-Wyart, F. & Quere, D. *Capillarity and Wetting Phenomena* (Springer, 2002).

[29] Sloutskin, E., Bain, C. D., Ocko, M. & Deutsch, M. Surface freezing of chain molecules at the liquid – liquid and liquid – air interfaces. *Faraday Discussions* 339–352 (2005).

[30] Baroud, C. N., Delville, J.-p., Gallaire, F. & Wunenburger, R. Thermocapillary valve for droplet production and sorting. *Physical Review E* **75**, 046302 (2007).

[31] Robert de Saint Vincent, M. *et al.* Laser switching and sorting for high speed digital microfluidics. *Applied Physics Letters* **92**, 154105 (2008).

[32] Ruckenstein, E. Can phoretic motions be treated as interfacial tension gradient driven phenomena? *Journal of Colloid and Interface Science* **83**, 77–81 (1981). URL https://linkinghub.elsevier.com/retrieve/pii/0021979781900114.

[33] Happel, J. & Brenner, H. *Low Reynolds Number Hydrodynamics* (Springer, Englewood Cliffs, 1983).

[34] Tailleur, J. & Cates, M. E. Statistical Mechanics of Interacting Run-and-Tumble Bacteria. *Physical Review Letters* **100**, 218103 (2008). URL https://link.aps.org/doi/10.1103/PhysRevLett.100.218103. abs/0803.1069.

[35] Fily, Y. & Marchetti, C. M. Athermal Phase Separation of Self-Propelled Particles with No Alignment. *Physical Review Letters* **108**, 235702 (2012). URL https://link.aps.org/doi/10.1103/PhysRevLett.108.235702.

[36] Redner, G. S., Hagan, M. F. & Baskaran, A. Structure and Dynamics of a Phase-Separating Active Colloidal Fluid. *Physical Review Letters* **110**, 055701 (2013). URL https://link.aps.org/doi/10.1103/PhysRevLett.110.055701.

[37] Stenhammar, J., Wittkowski, R., Marenduzzo, D. & Cates, M. E. Activity-Induced Phase Separation and Self-Assembly in Mixtures of Active and Passive Particles. *Physical Review Letters* **114**, 018301 (2015). URL https://link.aps.org/doi/10.1103/PhysRevLett.114.018301. 1408.5175.

[38] Cates, M. E. & Tailleur, J. Motility-Induced Phase Separation. *Annual Review of Condensed Matter Physics* **6**, 219–244 (2015).

[39] Solon, A. P. *et al.* Pressure is not a state function for generic active fluids. *Nature Physics* **11**, 673–678 (2015). abs/1412.3952.

[40] Jeanneret, R., Pushkin, D. O., Kantsler, V. & Polin, M. Entrainment dominates the interaction of microalgae with micron-sized objects. *Nature Communications* **7**, 12518 (2016). URL http://www.nature.com/articles/ncomms12518. abs/1602.01666.

[41] Kardar, M. *Statistical Physics of Particles* (Cambridge University Press, Cambridge, 2007).

[42] Ray, D., Reichhardt, C. & Reichhardt, C. J. O. Casimir effect in active matter systems. *Physical Review E* **90**, 013019 (2014). URL https://link.aps.org/doi/10.1103/PhysRevE.90.013019. 1402.6372.

[43] Harder, J., Mallory, S. A., Tung, C., Valeriani, C. & Cacciuto, A. The role of particle shape in active depletion. *Journal of Chemical Physics* **141** (2014). 1407.6743.

[44] Smallenburg, F. & Löwen, H. Swim pressure on walls with curves and corners. *Physical Review E* **92**, 032304 (2015). URL `https://link.aps.org/doi/10.1103/PhysRevE.92.032304`. `1504.05080`.

[45] Wensink, H. H., Kantsler, V., Goldstein, R. E. & Dunkel, J. Controlling active self-assembly through broken particle-shape symmetry. *Physical Review E* **89**, 010302 (2014). URL `https://link.aps.org/doi/10.1103/PhysRevE.89.010302`.

[46] Grosberg, A. Y. U. & Joanny, J. F. Nonequilibrium statistical mechanics of mixtures of particles in contact with different thermostats. *Physical Review E - Statistical, Nonlinear, and Soft Matter Physics* **92**, 1–10 (2015).

[47] Weber, S. N., Weber, C. A. & Frey, E. Binary Mixtures of Particles with Different Diffusivities Demix. *Physical Review Letters* **116**, 058301 (2016). URL `https://link.aps.org/doi/10.1103/PhysRevLett.116.058301`. `abs/1505.00525`.

[48] Altshuler, E. *et al.* Symmetry Breaking in Escaping Ants. *The American Naturalist* **166**, 643–649 (2005). URL `http://www.journals.uchicago.edu/doi/10.1086/498139`.

[49] Gelblum, A. *et al.* Ant groups optimally amplify the effect of transiently informed individuals. *Nature Communications* **6**, 7729 (2015). URL `http://www.nature.com/articles/ncomms8729`.

[50] Attanasi, A. *et al.* Information transfer and behavioural inertia in starling flocks. *Nature Physics* **10**, 691–696 (2014). URL `http://www.nature.com/articles/nphys3035`.

[51] Katz, Y., Tunstrom, K., Ioannou, C. C., Huepe, C. & Couzin, I. D. Inferring the structure and dynamics of interactions in schooling fish. *Proceedings of the National Academy of Sciences* **108**, 18720–18725 (2011). URL `http://www.pnas.org/cgi/doi/10.1073/pnas.1107583108`.

[52] Seeman, N. C. *Structural DNA Nanotechnology* (Cambridge University Press, 2015).

[53] Ben Zion, M. Y. *et al.* Self-assembled three-dimensional chiral colloidal architecture. *Science* **358**, 633–636 (2017).

[54] Zhu, G. *et al.* Microchemomechanical devices using DNA hybridization. *Proceedings of the National Academy of Sciences* **118**, e2023508118 (2021). URL `http://www.pnas.org/lookup/doi/10.1073/pnas.2023508118`.

[55] Ben Zion, M. Y., Modin, A., Caba, Y. & Chaikin, P. M. Cooperation in a fluid swarm of fuel-free micro-swimmers. *figshare* (2021). URL `https://doi.org/10.6084/m9.figshare.16559733.v1`.

[56] Ben Zion, M. Y., Modin, A., Caba, Y. & Chaikin, P. M. Cooperation in a fluid swarm of fuel-free micro-swimmers. *Arxiv cond-mat.soft* (2020). URL `http://arxiv.org/abs/2012.15087`. `2012.15087`.

[57] Mustafaev, R. A. Thermal conductivity of higher saturated n-hydrocarbons over wide ranges of temperature and pressure. *Journal of Engineering Physics* **24**, 465–469 (1973).

[58] Calado, J. C., Fareleira, J. M., Nieto de Castro, C. A. & Wakeham, W. A. Thermal conductivity of five hydrocarbons along the saturation line. *International Journal of Thermophysics* **4**, 193–208 (1983).

[59] Tanaka, Y., Itani, Y., Kubota, H. & Makita, T. Thermal conductivity of five normal alkanes in the temperature range 283-373 k at pressures up to 250 MPa. *International Journal of Thermophysics* **9**, 331–350 (1988).

[60] Schmidt, R. *et al.* Hydrocarbons. In *Ullmann's Encyclopedia of Industrial Chemistry*, 1–74 (Wiley-VCH Verlag GmbH & Co. KGaA, Weinheim, Germany, 2014). URL `http://doi.wiley.com/10.1002/14356007.a13{_}227.pub3`.

[61] Rueden, C. T. *et al.* ImageJ2: ImageJ for the next generation of scientific image data. *BMC Bioinformatics* **18**, 529 (2017).

[62] Allan, D. *et al.* soft-matter/trackpy: Trackpy v0.4.2 (2019). *Online repository* URL `https://doi.org/10.5281/zenodo.3492186`.

[63] Liberzon, A. *et al.* OpenPIV/openpiv-python: OpenPIV - Python (v0.22.2) with a new extended search PIV grid option (2020). URL https://doi.org/10.5281/zenodo.3930343.

[64] Oppenheimer, N., Stein, D., Ben Zion, M. Y. & Shelly, M. J. Hyperuniformity and phase enrichment in vortex and rotor assemblies. *Arxiv cond-mat.soft* (2021). URL http://arxiv.org/abs/2103.00296. 2103.00296.

[65] Stein, D. rotor_hyperuniformity. *gitHub repository* (2021). URL https://github.com/dbstein/rotor_hyperuniformity.

[66] Harper, M. python-ternary: Ternary Plots in Python. *Zenodo 10.5281/zenodo.594435* (2019). URL https://github.com/marcharper/python-ternary.
