# Supplementary material for: Cooperation in a fluid swarm of fuel-free micro-swimmers
Source: Nat Commun. 2022 Jan 10;13:184. doi: 10.1038/s41467-021-27870-9 (PMC8748659; doi:10.1038/s41467-021-27870-9)
Supplement: Supplementary file 3 — Description for Additional Supplementary Files [file 41467_2021_27870_MOESM3_ESM.pdf]

Description of additional Supplementary Data files

Supplementary Video 1 - Swimmer's activity inside the dense phase.

Supplementary Video 2 - Entrainment mediated corralling.

Supplementary Video 3 - Quick dispersing at low density.

Supplementary Video 4 - Schooling and anti-schooler at intermediate density.

Supplementary Video 5 - Internal flow inside the dense phase.

Supplementary Video 6 - Corralling of passive particle
